# Moss PIEZO homologs have a conserved structure, are ubiquitously expressed, and do not affect general vacuole function

**DOI:** 10.1080/15592324.2021.2015893

**Published:** 2021-12-24

**Authors:** Ivan Radin, Ryan A. Richardson, Elizabeth S. Haswell

**Affiliations:** a Department of Biology, MSC 1137‐154‐314, Washington University, St. Louis, MO USA; b NSF Center for Engineering Mechanobiology

**Keywords:** PIEZO, mechanosensitive ion channel, moss, *Physcomitrium*, vacuole

## Abstract

The PIEZO protein family was first described in animals where these mechanosensitive calcium channels perform numerous essential functions, including the perception of light touch, shear, and compressive forces. PIEZO homologs are present in most eukaryotic lineages and recently we reported that two PIEZO homologs from moss *Physcomitrium patens* localize to the vacuolar membrane and modulate its morphology in tip-growing caulonemal cells. Here we show that predicted structures of both *Pp*PIEZO1 and *Pp*PIEZO2 are very similar to that of mouse Piezo2. Furthermore, we show that both moss *PIEZO* genes are ubiquitously expressed in moss vegetative tissues and that they are not required for normal vacuolar pH or intracellular osmotic potential. These results suggest that moss PIEZO proteins are widely expressed mechanosensory calcium channels that serve a signaling rather than maintenance role in vacuoles.

## Introduction

**Figure 1.**
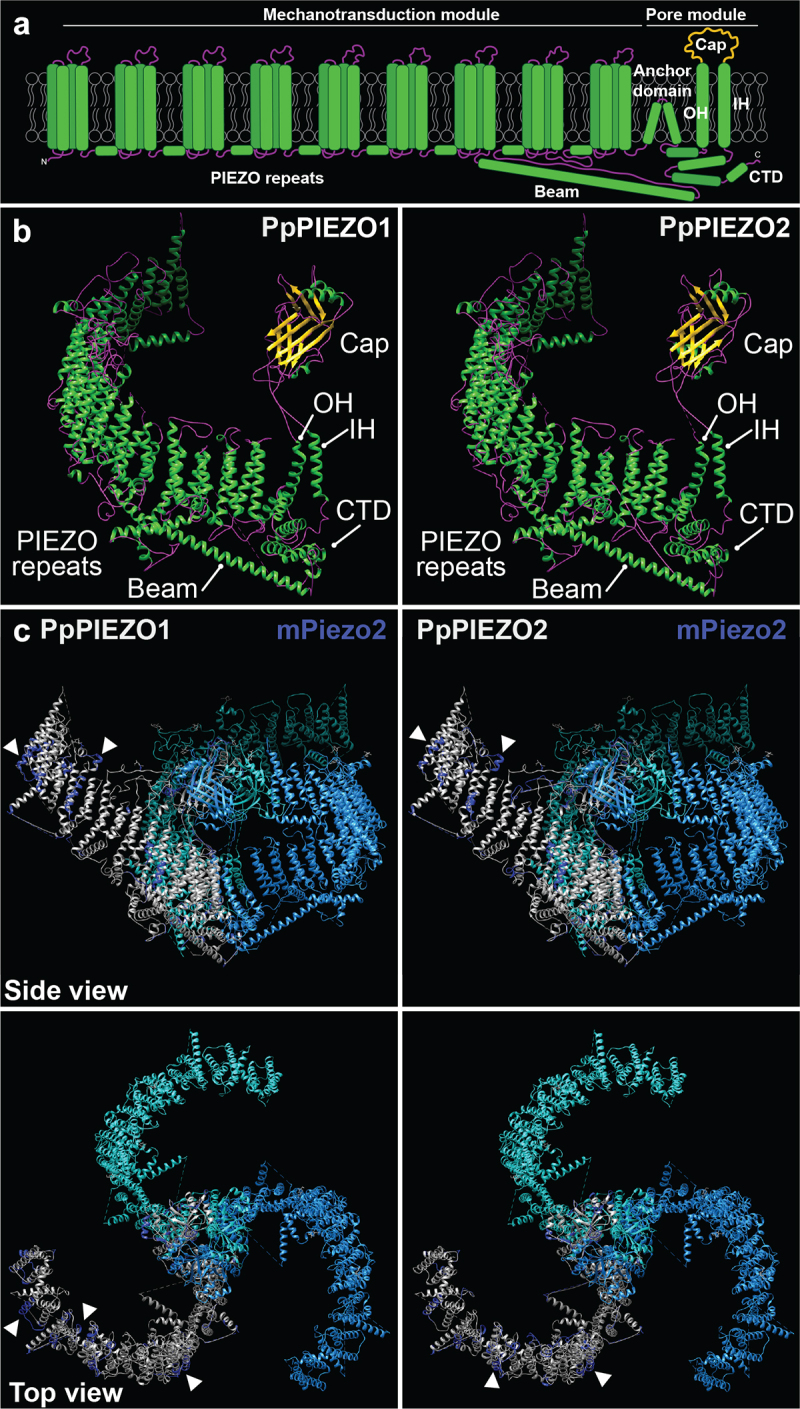
Models of PpPIEZO1 and PpPIEZO2 predicted protein structures. (a) Diagram of a typical PIEZO monomer, based on cryo-EM structures of mPiezo1 and mPiezo2. Not drawn to scale. OH, outer helix; IH, inner helix; CTD, C-terminal domain. (b) Phyre2-generated models of PpPIEZO1 and PpPIEZO2 monomers. Helices, strands/sheets, and coils are depicted in green, yellow, and magenta, respectively. (c) Predicted models of PpPIEZO1 and PpPIEZO2 monomers (gray) superimposed onto one subunit of the mPiezo2 homotrimeric complex (dark blue). The other two subunits of the mPiezo2 complex (light blue and cyan) are shown without the overlay. Models were visualized with UCSF Chimera software.^12^

Mechanosensitive ion channels are one of the main mechanisms by which cells perceive mechanical forces. In response to lateral membrane tension, these channels open and release ions down their electrochemical gradients.^1–3^The PIEZO protein family was first described in animals^4^ and has been implicated in the perception of light touch, shear stress, compressive forces, nociception, and other mechanostimuli. Animal PIEZO channels are embedded in the plasma membrane and conduct cations, including calcium.^4–6^ Cryo-EM structures of two mouse homologs showed that animal PIEZOs form large propeller-shaped complexes comprised of three identical subunits.^7–11^ Each subunit has 38 transmembrane domains, the last two of which are called the inner and outer helix and form the pore module (Figure 1a). The pore module also includes the cap domain, which connects the inner and outer helix and sits above the pore, the anchor domain, and the C-terminal domain. The first 36 transmembrane domains of each monomer are organized in 9 consecutive PIEZO repeats to form the mechanotransduction module. The beam domain connects the mechanotransduction module to the pore module.^7–11^

While both the structure and functions of animal PIEZOs are well-described, little is known about their homologs in plants. We recently reported that two PIEZO homologs from the moss *Physcomitrium patens* (*Pp*PIEZO1 and *Pp*PIEZO2) are required for normal growth and cytoplasmic calcium oscillations in the tip-growing moss caulonemal cells.^13^ Surprisingly, moss PIEZOs localized to the vacuolar membrane, in contrast to their animal counterparts. Plant vacuoles are large aqueous organelles with numerous functions, including storage of ions and metabolites, maintenance of cellular osmotic potential and pH, and degradation.^14^ Vacuolar morphology is highly cell-type specific and dynamic, often changing in response to external or internal cues.^15^ Moss PIEZOs may be involved in this process, as the disruption of *Pp*PIEZO1/2 led to a dramatic expansion of tubule-like vacuoles normally seen in WT caulonemal cells. This vacuolar localization and function appear to be conserved among land plant PIEZOs, as we observed a similar vacuolar expansion in pollen tubes from *Arabidopsis thaliana* lines lacking the single PIEZO homolog *At*PIEZO1.^13^ *At*PIEZO1 has also been implicated in the suppression of systemic viral spread^16^ and mechanotransduction in the root tip,^17,18^ and a chimera containing the pore of *At*PIEZO1 within the mPIEZO1 sequence has mechanosensitive ion channel activity.^19^ Here we further explore the predicted structure, tissue-specific expression patterns, and physiological roles of moss PIEZO homologs.

## Predicted structures of *Pp*PIEZO1 and *Pp*PIEZO2

We used Phyre2^19^ to predict the structures of *Pp*PIEZO1 and *Pp*PIEZO2 using the cryo-EM structure of mPiezo2 (6KG7 model from^7^) as a template. For both proteins, 67% of the sequence was modeled. In the models, the main structures of the pore domain^7^ (Figure 1a) can be identified: cap, beam, inner and outer helix, and C-terminal domain (Figure 1b). Furthermore, the transmembrane domains that comprise the PIEZO repeats within the mechanotransduction module are organized into the characteristic propeller blade shape of other PIEZO channels.^7,9^

While there is little conservation of primary protein sequence between moss and mouse PIEZO homologs (20% identity and 34% similarity for full-length *Pp*PIEZO1 and mPiezo2 and 20% identity and 33% similarity for full-length *Pp*PIEZO2 and mPiezo2; calculated with EMBOSS Needle^20^) (Fig. S1), we wanted to assess their structural similarities. To this end, we superimposed the Phyre2 models of *Pp*PIEZO1/2 monomers onto the mPiezo2 homotrimeric complex cryo-EM structure.^7^ As shown in Figure 1c, the overall structures of moss and mouse PIEZO homologs are well conserved, with an almost complete overlap in the pore module. In the mechanotransduction module, a substantial overlap can also be observed. However, in a few regions along the blade (Figure 1c, arrowheads) some discrepancies between mouse and moss PIEZOs can be seen.

While using mPiezo2 as a template for modeling of *Pp*PIEZO1/2 likely accounts for some of the similarities between the structures, these models show that moss PIEZO homologs could achieve a very similar organization to the animal ones. To provide further support for this idea, we used the AlphaFold2 program,^21,22^ to predict the structures of the C-terminal regions of mPiezo2, *Pp*PIEZO1 and *Pp*PIEZO2 (this region corresponds to the pore module and the last PIEZO repeat in mammalian PIEZO homologs). In all three cases, very similar structures with easily identifiable domains characteristic of PIEZO channels were obtained (Fig. S2).

This evidence for structural conservation between plant and animal homologs suggests that plant PIEZOs also function as mechanosensitive calcium channels, analogous to their animal counterparts. This is in line with our previous finding that moss PIEZOs are properly oriented within the vacuolar membrane to release calcium from the vacuolar stores into the cytosol,^13^ as well as with a recent report from Mousavi et al.^18^ that a chimeric *Arabidopsis*/mouse PIEZO channel can conduct calcium in response to mechanical force. We further note that the predicted full-length structures of several flowering plant PIEZO homologs present in the AlphaFold Database show a striking resemblance to animal PIEZO cryo-EM structures^7–11^ (alphafold.ebi.ac.uk/search/text/PIEZO).

## Expression pattern of *PpPIEZO1* and *PpPIEZO2* in moss vegetative tissues

**Figure 2.**
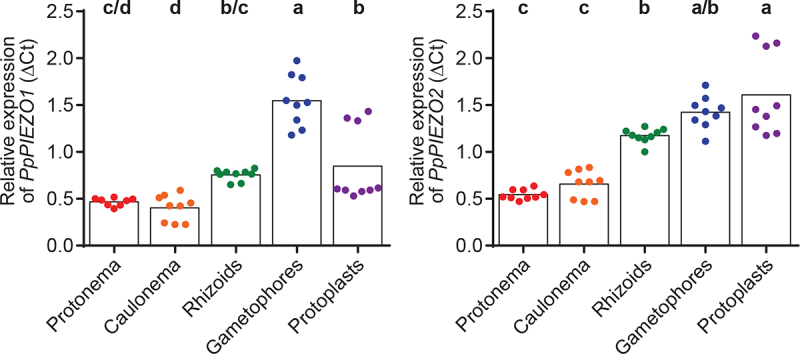
*Pp*PIEZO1 and *Pp*PIEZO2 are ubiquitously expressed in moss vegetative tissues. Relative expression of *PpPIEZO1* (left) and *PpPIEZO2* (right) in various moss vegetative tissues. C_t_ values were normalized to the geometric mean of two housekeeping genes (Pp3c8_16590 and Pp3c14_7550). Datapoints shown are from three biological replicates, three technical replicates each (except for *PpPIEZO1* protonema, where one outlier technical triplicate was removed from the second biological replicate). Bars, average values. Statistics, one-way ANOVA with Post-Hoc Tukey’s test (*p* < .05). Letters denote grouping based on statistical differences.

In our previous work,^13^ we focused on the function of *Pp*PIEZOs in caulonemal cells. The publicly available microarray dataset available on the Physcomitrella eFP Browser suggested their ubiquitous expression in all moss tissues. Here we set out to validate such a broad expression pattern by isolating RNA from protonemal cells, caulonemal cells, rhizoids, gametophores, and protoplasts and determining *PpPIEZO1* and *PpPIEZO2* expression levels using qPCR. As can be seen in Figure 2, mRNA transcripts for both *PpPIEZO1* and *PpPIEZO2* were detected in all tissues tested. Levels of *PpPIEZO1* transcripts were comparable in all tissues, except in gametophores where higher transcript levels were detected. *PpPIEZO2* transcript levels were lower in juvenile tissue (protonema and caulonema) compared to mature tissues (gametophores and rhizoids) and protoplasts. We did not test expression in the reproductive organs.

Both *PpPIEZO* transcripts accumulated to significantly higher levels in gametophore cells than in caulonemal cells, where *Pp*PIEZOs play an important role in the modulation of vacuolar morphology.^13^ As gametophore cells grow through cell expansion, these results suggest that *Pp*PIEZO channel function is not limited to tip-growing caulonemal cells. Higher *Pp*PIEZO expression could be an adaptation to cell expansion-based growth, as comparted to tip growth, or could be linked to a specific need associated with vacuolar morphology or mechanical signaling in this cell type. Future experiments to examine the localization of PIEZO1 and PIEZO2 in these tissues, and to understand the effect of PIEZO1 and PIEZO2 mutations on vacuolar morphology and on the mechanical responsiveness of gametophytic tissue will help address these questions.

## Vacuolar pH and osmotic potential in *PpPIEZO* knock-out moss lines

**Figure 3.**
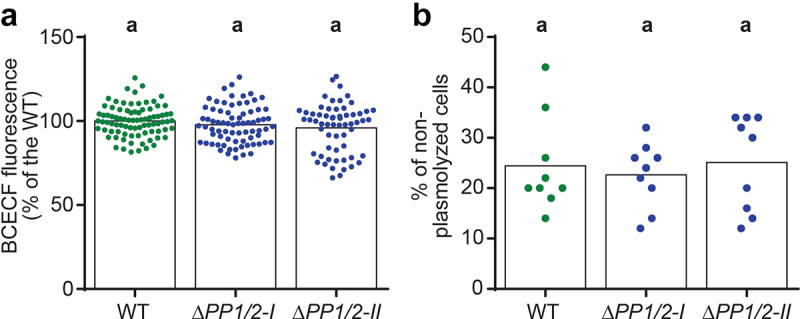
*Pp*PIEZO1/2 mutants have normal vacuolar pH and intracellular osmotic potential. (a) Ratio of BCECF fluorescence after excitation with 488 and 445 nm light. Datapoints shown are individual cells from six experiments, each normalized to their respective WT average (N = 84, 75, and 61 cells for WT, *ΔPP1/2-I*, and *ΔPP1/2-II*, respectively). Statistics, Kruskal-Wallis test with Dunn’s multiple comparisons test (*P* < .05). (b) Percentage of non-plasmolyzed apical caulonemal calls after 5 min exposure to BCDAT media supplemented with 350 mM mannitol. Datapoints shown are from three independent experiments. Each data point corresponds to the percentage calculated from 50 cells from a single plant. Bars, average values. Statistics, as in (A).

Our previous work showed that deletion of moss PIEZO homologs leads to altered growth and dramatic expansion of the vacuoles in the tip region of the apical caulonemal cells.^13^ We next wondered whether deletion of *Pp*PIEZOs might affect vacuolar function as well as morphology. As mentioned above, vacuoles play a role in the maintenance of pH homeostasis and are characterized by a lower luminal pH than the surrounding cytoplasm.^14^ We therefore tested whether *Pp*PIEZO1/2 double mutants (*ΔPP1/2*) have different vacuolar pH than the WT by staining cells with the pH-sensitive dye 2′,7′-bis-(Carboxyethyl)-5-(and-6)-carboxyfluorescein (BCECF). We imaged BCECF-stained vacuoles in apical caulonemal cells and found no difference in 488/445 nm ratio values between WT and *ΔPP1/2* cells (Figure 3a). This shows that *ΔPP1/2* cells are able to maintain normal vacuolar pH, despite striking changes in their morphology. It thus appears that *Pp*PIEZO activity does not affect the systems important for vacuolar pH maintenance.

Vacuoles also participate in the maintenance of intracellular osmotic potential, so we tested whether WT and *ΔPP1/2* cells differ in their osmotic potential. In cells with osmotic potential lower than that of the environment, the protoplast (cell content) will lose water, shrink, and detach from the cell wall in a process known as plasmolysis. On the other hand, in non-plasmolyzed cells, the osmotic potential is equal to or great than that of the environment. Thus, the likelihood of plasmolysis under hyperosmotic stress is often used as a proxy for relative intracellular osmotic potential.^23^ We observed no significant difference in the percentage of non-plasmolyzed cells in 350 mM mannitol between WT and *ΔPP1/2* caulonemal cells (Figure 3b), suggesting that the altered vacuolar morphology in *ΔPP1/2* mutants does not affect their ability to maintain intracellular osmotic potential. These results also indicate that the observed growth defects in *ΔPP1/2* mutant caulonemal cells^13^ cannot be attributed to differences in osmotic potential. Instead, it may be that the changes in cytosolic Ca^2+^ oscillatory profiles observed in *ΔPP1/2*^13^ affect growth processes, or that the expanded vacuoles found in these cells prevent the proper spatial assembly of the cytoskeleton or other cellular elements.

## Summary

The findings presented here supplement our recent publication,^13^ and improve our understanding of PIEZO homologs in moss. *Pp*PIEZOs have similar predicted structures as mPiezo2, providing support for a conserved molecular function as mechanosensitive cation channels. The fact that *Pp*PIEZOs are ubiquitously expressed in vegetive tissues indicates that PIEZOs likely have functions beyond those already described in caulonemal cells, yet to be discovered. Finally, *Pp*PIEZOs modulate the vacuolar morphology in caulonemal cells without affecting pH or osmotic potential. Altogether, these data suggest a model wherein moss PIEZOs function as mechanosensitive calcium channels that serve to modulate vacuolar morphology through signaling, rather than through general maintenance of vacuolar functions.

## Methods

### Tissue sampling and QPCR

Moss was cultured as previously described.^13^ Protonemal cell samples were collected from plants cultured on cellophaned BCDAT media for 5 days after grinding. Caulonemal cells were manually cut and separated from dark-grown (described in^13^) plants. Rhizoids and gametophores were collected from 5-week-old plants cultured on uncellophaned BCD plates (without ammonium-tartrate) under a standard light cycle. Gametophores together with rhizoids were pulled from the media and the two were separated with a scalpel. The middle section connecting the two tissue types was discarded. The excess water in the tissue was removed by squeezing between paper wipes, before freezing in liquid nitrogen. Protoplasts were isolated from 5-day-old protonema tissue as previously described.^13^ Total RNA from each sample was isolated with a RNeasy Plant Mini Kit and on-column DNase treatment (Qiagen). 100 ng of RNA was then used to synthesize cDNA (Oligo(dT) primer) with Superscript IV (ThermoFisher).

For qPCR reactions, approximately 2 ng of cDNA was mixed with 2x PowerUp SYBR Green Master Mix (Applied Biosystems) and primers (Supplementary Table 1). Reactions were run on the StepOnePlus system from Applied Biosystems (95°C for 10 min; 40 cycles of 95°C for 15 s and 60°C for 1 min; followed by a melt curve). *PpPIEZO1* and *PpPIEZO2* transcript levels were normalized to the geometric mean of two housekeeping genes (Pp3c8_16590 and Pp3c14_7550;^24^ Supplementary Table 1) using the ΔCt method.

## BCECF staining and imaging

To evaluate vacuolar pH, 6-day-old WT and *Pp*PIEZO1/2 double mutant (*ΔPP1/2*) plants (started from fragmented protonema, see^13^ for details) were stained with BCECF as described in^25^ using liquid BCDAT media. Plants were mounted onto a coverslip at the bottom of a 35 mm Petri dish as described in^13^ and imaged with an inverted Olympus FV3000 confocal microscope (objective UPLSAPO 60xW NA1.2). BCECF was excited with 445 and 488 nm, and in both cases, emission was collected in the 500–550 nm range. The 488/445 nm ratio (after background subtraction) is proportional to the luminal pH (higher ratio = higher pH).

## Plasmolysis quantification

6-day-old WT and *ΔPP1/2* plants (started from fragmented protonema) were mounted onto a coverslip at the bottom of a 35 mm Petri dish (see^13^ for details) and covered with 3 mL of BCDAT media supplemented with 350 mM mannitol. After a 5 min incubation, plants were imaged using brightfield and UPLSAPO 60xW NA1.2 objective. For each plant, 50 apical caulonema cells were evaluated for signs of plasmolysis (visible separation of the protoplast from the cell wall at the tip).

## Supplementary Material

Supplemental Material
