# Supplementary material for: Moss PIEZO homologs have a conserved structure, are ubiquitously expressed, and do not affect general vacuole function
Source: Plant Signal Behav. 2021 Dec 24;17(1):2015893. doi: 10.1080/15592324.2021.2015893 (PMC8920221; doi:10.1080/15592324.2021.2015893)
Supplement: Supplemental Material [file KPSB_A_2015893_SM8927.pdf]

```
                                   TM33                                     TM34
mPiezo2       TDVYVLMFLADTVDFIIIVFGFWAFGKHSAAADITSSLSEDQVPGPFLVMVLIQFGTMVV   2318
PpPIEZO1      VDLYAYIFGTDLLAFLFVALVYQSFVKHSPKL-LDVTRVEDQFPKDFIIVLMTLFFMIVA   2080
PpPIEZO2      VDLYAYIFGTELLTFVFVALFYQPLMKHSSGL-FDVTQVEDQFPKGFIIVLMTLFFLIVV   2080
              .*:*. :* :: : *::::.: : : ***     :  :  ***.*  *::::: *  :*.

                                          TM35                         TM36
mPiezo2       DRALYLRKTVLGKVIFQVILVFGI------HFWMF--FILPGVTERKFSQNLVAQLWYFV   2370
PpPIEZO1      DRVLYLCSFATGKMLSYFFSLVLYTTYASKVVWEIEATIQPESP-NYFLQLLPLRLFYLM   2139
PpPIEZO2      DRVLYLCSFASGKVIYYLCTLMLYTGYVSQFVWSIEDHETVEAKNKHEFRLLPLRIFYMM   2140
              **.*** . . **::  .  :.          .* :           .   : *  :::*::

                                                    Anchor-α1     Anchor-α2
mPiezo2       KCVYFGLSAYQIRCGYPTR--VLGNFLTKSYNYVNLFLFQGFRLVPFLTELRAVMDWVWT   2428
PpPIEZO1      KGLSLALQASQIKYGLPHKSALYGQFLARRVNALSWCGYRFYRAVPFLFELRCVLDWSCT   2199
PpPIEZO2      KALSLALQAFQIKYGLPHKSALYGQFLARKVNLMSWNCFRLYRALPFLFELRCVLDWSCT   2200
              * : :.*.* **: * * :  : *:**:: * :.  :: :* :*** ***.*:**  *

                          Anchor-α3                          TM37/OH
mPiezo2       DTTLSLSSWICVEDIYAHIFILKCWRESEKRYPQPRGQKKKKAVKYGMGGMIIVLLICIV   2488
PpPIEZO1      TTALNMYDWLKLEDIYGSLFLVQCDVKLV-RARHLLGQKQGVWIKFCSGILLFCVLIGVI   2258
PpPIEZO2      TTALNMYDWLKLEDIYGSLFLVQCDNKLN-REKHRLGEKQSMFIKFCSGVLLFAILIAVI   2259
               *:*.: .*: :****. :*:::*  :   *  :  *:*:   :*:  * ::: :** ::

                                                                     Cap-α1
mPiezo2       WFPLLFMSLIKSVAGVINQPLDVSV---TITLGGYQPIFTMSAQQSQLKVMDNSKYNEFL   2545
PpPIEZO1      WAPMLIYSNGNPT-NMANPVNDVRGGIEVKTVGGKFPLYETGLC-----------HIFNL   2306
PpPIEZO2      WAPMLIYSSGNPT-NTPNLVMDVHAGIAVKTAGGVFKLYETGLC-----------HNHTL   2307
              * *:*: *  : . .  *   **     . * **  ::  .                :  *

                    Cap-α2                        Cap-α3
mPiezo2       KSFGPNSGAMQFLENYEREDVTVAELEGNSNSLWTISPPSKQKMIQELTDPNSCFSVVFS   2605
PpPIEZO1      ES--PPKDALSTLSAYDIRDIQIVCCEPDAAALWQIPPSTLQNLIKSIHDGDLSFYAWWQ   2364
PpPIEZO2      TGTRYPDEHRSVLAGYDPRDVQVICCEPDGASLWLIPPSTLKSLIHSIDSDDLVFSSWWD   2367
               .    .   . *  *: .*: :   * :. :** * * : :.:*:.: . :  *   :.

                                    Cap-α4
mPiezo2       WSIQRNMTLGAKAEIATDKLSFPLAVATRNSIAKMIAGNDTESSNTPVTIEKIYPYYVKA   2665
PpPIEZO1      F--NRERPKGKELALWSED---VTSVDEDLFGGGSQLRAVLNGTLASVNIAKLYPLYFRV   2419
PpPIEZO2      F--HRERPKEKELASYALQ---PQQ---DKNVLPDQLKAVLNGTSNSIYIDKLYPQYFRV   2419
              :  :*:     :    : .                          :.:   : * *:** *.:.

mPiezo2       PSDSNSKPIKQLLSENNFMNITIILFRDNVTKSNSEWWVLNLTGSRIFNQGSQALELVVF   2725
PpPIEZO1      PGSGEVRALET---ESLRVSGNLTL----NKEQGQAWWSFLRENPSPDDGCGKMLGPIAY   2472
PpPIEZO2      PSSGDVHILEG---SNVYISGNLTL----KREFGQAWWSFESDI-SLANDCGSMMGPAAI   2471
              *...: : ::    ..  :. .: *      : .. ** :           :  .. :   .

                                   TM38/IH
mPiezo2       --NDKVSPPSL--GFLAGYGIMGLYASVVLVIGKFVREFFSGISHSIMFEELPNVDRILK   2781
PpPIEZO1      VVSEEVPPKGLLGETLSKFSIWSLYITFVLAVGRFIRLQCSDIRMRIPYENFPACDRLVA   2532
PpPIEZO2      TVSEEVPPKGLLGETLSRFSIWSLYITFVLAVGRFIRLQCADIRMRIPYENFPACDRLVA   2531
                .::* * .*    *: :.* .** :.**.:*:*:*   :.*   * :*::*  **::

                CTD-α1          CTD-α2        CTD-α3
mPiezo2       LCTDIFLVRETGELELEEDLYAKLIFLYRSPETMIKWTREKTN                    2824
PpPIEZO1      ICEDIYAARAEGELELEEGLFWTIVKIYRAPYMLMEYTKVE--                    2573
PpPIEZO2      ICEDIYAARAAGELELEEGLFWTLIKIYRAPYILMEYTKVE--                    2572
              :* **: .*  *******.*: .:: :**:*  ::::*: :
```

Figure S1. Alignment of PIEZO C-terminal region protein sequences.
Protein sequences from mouse Piezo2 and moss PIEZO1 and 2 C-terminal regions, aligned with Clustal Omega [19]. Secondary structures based on the mPiezo2 cryoEM model [7]) are indicated (helices, green; strands/sheets, yellow). The names of α-helixes reflect the domains to which they are attributed to. TM, transmembrane domain; OH, outer helix; IH, inner helix; CTD, C-terminal domain.

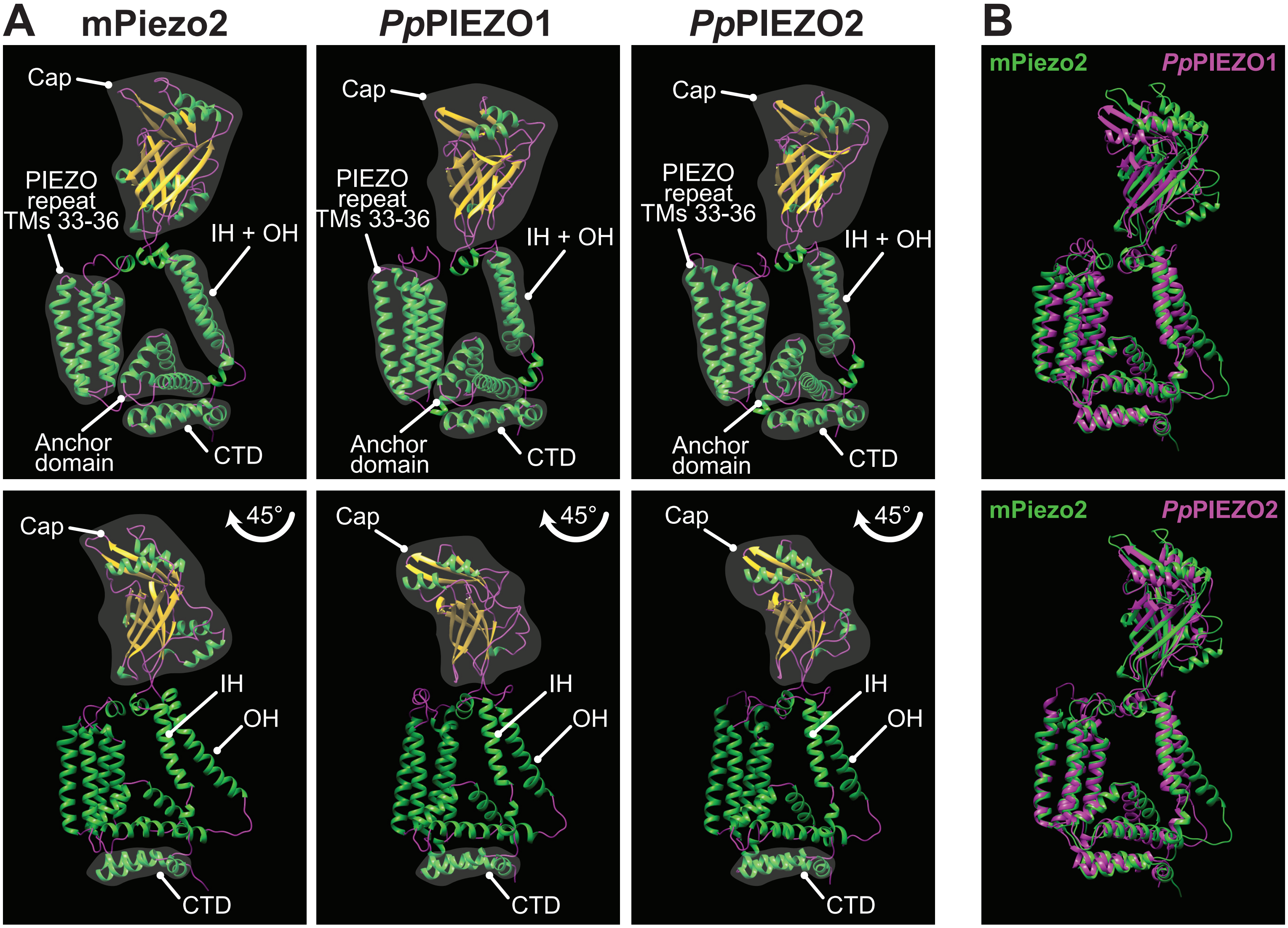

Figure S2. AlphaFold2 models of PIEZO domains.
(**A**) AlphaFold2-generated relaxed models of PIEZO domains from mouse Piezo2 and moss PIEZO1 and 2. Helices, strands/sheets, and coils are depicted in green, yellow, and magenta, respectively. Models in the lower panels were rotated approximately 45° around the Z-axis respective to the models in the top panels. TM, transmembrane domain; OH, outer helix; IH, inner helix; CTD, C-terminal domain. The exact sequences used for the AlphaFold2 modeling are given in Fig. S1. (**B**) Models of PIEZO domains from *Pp*PIEZO1 (top) and *Pp*PIEZO2 (bottom), in magenta, superimposed onto a model from mouse Piezo2, in green. Models were visualized with UCSF Chimera software [25].

Supplementary Table 1

Locus	Gene	Sequence (5’ to 3’)	Concentration (μM)	Efficiency (%)	Amplicon size (bp) cDNA	Amplicon size (bp) gDNA	Reference
Pp3c8_16590V3.1	Adenine phosphoribosyltransferase	F: AGTATAGTCTAGAGTATGGTACCG<br>R: TAGCAATTTGATGGCAGCTC	0.25<br>0.25	105.9	131	131	Le Bail et al., 2013.
Pp3c14_7550V3.1	60S Ribosomal protein L31	F: ACGGACATTGCATTTAAGACCT<br>R: GTCGATTACCTGTGGAGAAGAC	0.25<br>0.25	103.0	211	211	Le Bail et al., 2013.
Pp3c9_13300V3.1	*PpPIEZO1*	F: TACTTTCGAGTACCTGGGTCTG<br>R: CATCTTGCCACATCCGTCATC	0.25<br>0.25	103.7	153	436	This paper
Pp3c3_17170V3.1	*PpPIEZO2*	F: GTACCAAGTTCTGGAGATGTGC<br>R: GAAACTGTAATGGCTGCTGGAC	0.25<br>0.25	102.0	167	417	This paper
